# Supplementary material for: Why is there a gap in self-rated health among people with hypertension in Zambia? A decomposition of determinants and rural‒urban differences
Source: BMC Public Health. 2024 Apr 12;24:1025. doi: 10.1186/s12889-024-18429-6 (PMC11015612; doi:10.1186/s12889-024-18429-6)
Supplement: Supplementary file 1 — Supplementary Material 1 [file 12889_2024_18429_MOESM1_ESM.pdf]

Association between health and independent variables using a Probit Model

```
Average marginal effects                    Number of obs     =      1,058
Model VCE    : OIM

Expression   : Pr(health_cat), predict()
dy/dx w.r.t. : Age 1.sex 1.edu 2.edu 3.edu HIV_Prev 2.region pcexpend 2.use_facili
```

	dy/dx	Delta-method Std. Err.	z	P>|z|	[95% Conf.	Interval]
Age	-.006424	.0008427	-7.62	0.000	-.0080756	-.0047724
1.sex	.0027808	.0340972	0.08	0.935	-.0640485	.0696102
edu						
Primary	.0830649	.0395677	2.10	0.036	.0055137	.1606162
Secondary	.1698239	.0468829	3.62	0.000	.0779352	.2617126
Tertiary	.2293833	.0709237	3.23	0.001	.0903753	.3683912
HIV_Prev	.0072812	.0037181	1.96	0.050	-6.15e-06	.0145686
region						
Urban	.0346074	.0320482	1.08	0.280	-.0282059	.0974206
pcexpend	-8.51e-06	5.92e-06	-1.44	0.150	-.0000201	3.09e-06
use_facility						
No	.0141714	.0307035	0.46	0.644	-.0460064	.0743492

Note: dy/dx for factor levels is the discrete change from the base level.
